# Structure, function and regulation of mammalian glucose transporters of the *SLC2* family

**DOI:** 10.1007/s00424-020-02411-3

**Published:** 2020-06-26

**Authors:** Geoffrey D. Holman

**Affiliations:** Department of Biology and Biochemistry, University of Bath, Bath, BA2 7AY UK

**Keywords:** Glucose transport, GLUT proteins, Membrane transport, GLUT1, GLUT2, GLUT3, GLUT4, GLUT5, Regulated transport, Insulin, Hypoxia, ATP depletion

## Abstract

The *SLC2* genes code for a family of GLUT proteins that are part of the major facilitator superfamily (MFS) of membrane transporters. Crystal structures have recently revealed how the unique protein fold of these proteins enables the catalysis of transport. The proteins have 12 transmembrane spans built from a replicated trimer substructure. This enables 4 trimer substructures to move relative to each other, and thereby alternately opening and closing a cleft to either the internal or the external side of the membrane. The physiological substrate for the GLUTs is usually a hexose but substrates for GLUTs can include urate, dehydro-ascorbate and myo-inositol. The GLUT proteins have varied physiological functions that are related to their principal substrates, the cell type in which the GLUTs are expressed and the extent to which the proteins are associated with subcellular compartments. Some of the GLUT proteins translocate between subcellular compartments and this facilitates the control of their function over long- and short-time scales. The control of GLUT function is necessary for a regulated supply of metabolites (mainly glucose) to tissues. Pathophysiological abnormalities in GLUT proteins are responsible for, or associated with, clinical problems including type 2 diabetes and cancer and a range of tissue disorders, related to tissue-specific GLUT protein profiles. The availability of GLUT crystal structures has facilitated the search for inhibitors and substrates and that are specific for each GLUT and that can be used therapeutically. Recent studies are starting to unravel the drug targetable properties of each of the GLUT proteins.

## Introduction

The *SLC* (SoLute Carrier) gene family has been classified into 65 sub-families with identities within the sub-family that differ more than 20–25% from other *SLC*s [38, 89]. The *SLC2* sub-families of 14 related genes are thus distinct from the closest relatives (which are the *SLC19* sub-family) and lie within the major facilitator superfamily (MFS) group which codes for proteins whose function is to facilitate membrane transport of substrates [89]. The 14 SLC2 genes code for proteins that are further sub-divided into phylogenetically distinct classes 1-3 of GLUT (glucose transport) proteins [106].

The proteins of the *SLC2* family have 12 transmembrane spans (TMs 1-12) with intracellular N- and C-termini. Signature motifs in the GLUTs include highly conserved salt bridging motifs RXGRR between cytoplasmic loop of TM2 and TM3, with a repeated of the salt bridge sequence between the cytoplasmic loop between TM8 and TM9. These motifs are present in all proteins of the MFS superfamily and are associated with catalytic conformational change that occurs in mediated transport. The proteins are thought to have evolved from 4 inverted trimer repeats as TM1-3 has some sequence similarity to an inverted TM4-6 and TM7-9 has some sequence similarity to an inverted TM10-12. Such inverted repeats are thought to emerge from gene duplication and fusion [75, 167]. There is also structural pseudo-symmetry in which TM1-6 (the N-terminal half) is mirrored by TM7-12 (the C-terminal half) with the 2 half proteins separated by a large cytoplasmic loop between TM6 and TM7 [161]. There are also sequence motifs that are highly conserved and unique to the GLUT family and these regions may play a role in recognition of hexose-like substrates [11].

All the GLUT proteins were originally presumed to catalyse hexose transport into and out of cells. This is clearly the case for the class 1 GLUT proteins. However, class 2 and class 3 GLUT proteins do not necessarily have a primary function in catalysing glucose transport. In many cases, they can be shown to catalyse glucose uptake in experimental systems. However, some of these proteins (particularly the class 3 group) have alternative substrates and therefore alternative functions within their physiological settings, and at an appropriate endogenous cellular location.

Much of the experimental work on the GLUT proteins has focussed on the mammalian glucose transporters that are most abundant. These GLUTs (GLUTs 1-4) catalyse (facilitate) passive movement of glucose down concentration gradients. These gradients are usually from the blood system to the cell interior, but in the case of the liver, these gradients can be from the cell to the systemic blood stream. Once transport down a concentration gradient has been achieved, then net flux into or out of the cell is zero, but transport of glucose continues by a process of equilibrium exchange. Glucose influx and efflux through the protein continue but these exchange fluxes are equal [67]. The GLUT family of transport proteins thereby cooperatively function to provide a supply of glucose in the direction needed for cell metabolic processes while maintaining a remarkably constant blood glucose level (5 mM after fasting in humans) [84, 154]. These glucose transporters are often rate limiting for subsequent metabolism [82] and thus provide a point at which metabolic flux can be controlled.

## Class 1: GLUTs 1, 2, 3, 4 and 14

Class1 GLUTs have an N-linked asparagine with varying chain length glycan polymers that often give a broad spread of the protein when detected by Western blotting after resolution on SDS-PAGE gels. The topography of the GLUTs, particularly GLUT1, has been explored by a wide range of scanning mutagenesis experiments [152] that have revealed regions of the protein that are essential for transport.

### GLUT1

The GLUT1 transporter is present in high amounts in human erythrocytes and because of the relative ease of working with these cells, it has been most studied from the structure related to function perspective [49, 152, 154]. The large amount of the protein (approximately 5% of the membrane protein) was an important factor in its purification. A peptide sequence was obtained and used to identify a cDNA clone and ultimately the DNA sequence [152]. GLUT1 is still the only endogenous GLUT that has been purified to homogeneity and which can be identified as a Coomassie-staining protein on an SDS-PAGE gel. Methods for purification of recombinant GLUT1 have gradually been improved over several decades and ultimately GLUT1 was crystallised in an inward facing conformation by the Yan group [56].

GLUT1 has a *K*_m_ for glucose influx (around 2 mM) which is significantly lower than blood glucose levels [16, 112, 187]. The kinetics of glucose and galactose uptake have been extensively studied and reveal that below physiological temperatures, the *K*_m_ and *V*_max_ for efflux can up to 10-fold higher than for influx. In addition, the kinetic parameters for exchange transport (*K*_m_ and *V*_max_) are both approximately 10-fold higher than for net influx at low temperatures. These data suggest that influx (but not efflux or exchange transport) can be rate limited by the availability of binding sites at the exofacial surface of the erythrocyte. The physiological importance of this property is unclear and indeed, the asymmetry of binding site availability is much less evident at physiological temperatures [27, 219]. However, the property may allow rapid glucose efflux from the erythrocyte when these cells reach conditions of relatively low plasma glucose concentrations. The transport kinetic properties associated with GLUT1 in erythrocytes may be specific for this cell type and the asymmetry has not been extensively studied in other cell types. GLUT1 has very low affinity for the ketoses fructose and sorbose with affinity constants in the molar range [14].

GLUT1 is very abundant at the endothelial cells of blood-brain barrier and this supply of glucose to the brain must always be efficient, even at the expense of depriving other tissues of glucose. In this location, it provides a rate limiting barrier for glucose reaching the brain [215]. Consistent with this hypothesis, GLUT1 levels at this barrier are increased when blood glucose concentrations are low and in clinical hypoglycaemia [191]. GLUT1 is also present at high levels in the endometrial stromal cells of the placenta and is essential for viability and survival of the foetus [76, 149].

GLUT1 is expressed in varying degrees in most human cells, often along with other class 1 glucose transporters, and its availability at the plasma membrane can be regulated by membrane protein translocation in tissues where glucose uptake is hormonally controlled [92]. Several stress stimuli, including osmotic shock, are known to increase GLUT1 levels and possibly GLUT1 catalytic activity [12, 15]. GLUT1 can bind ATP and other nucleotides and these interactions may have a regulatory role [42]. GLUT1 knockout is embryonically lethal [221] but GLUT1 haplo-insufficiencies (due to inherited GLUT1 mutations) lead to range of metabolic imbalances and symptoms that include epileptic seizures [36, 162]. The study of these naturally occurring mutations has aided the mapping of important regions and residues in GLUT1 that are necessary for transport [49, 95, 153].

### GLUT2

GLUT 2 is present at high levels in the liver, pancreatic β cells, the intestine and other cells of the endoderm lineage [210]. By contrast with GLUT1, it has relatively high affinity for both glucose and fructose with a *K*_m_ of 20 mM for glucose and *K*_m_ of 67 mM for fructose [43, 84]

Glucose and fructose enter the portal circulation via GLUT2-mediated transport, primarily at the basolateral border of intestinal epithelial cells. However, GLUT2 can also be translocated to the brush border of intestinal cells depending upon the dietary sugar load [117]. In the liver, GLUT2 avidly removes fructose from the portal vein so that only low levels of fructose are present in the systemic circulation [163]. GLUT2 on the basolateral borders of kidney epithelial cells is required for glucose reabsorption and GLUT2 knockout mice have elevated glucosuria [87]. GLUT2 has a *K*_m_ value of influx of approximately 20 mM which is higher than fasting blood glucose reflecting the functions of this protein in supplying glucose to the systemic circulation.

In the mouse [211] but not human [144] pancreas, GLUT2 is a key component (along with glucokinase and Kir6.2/SUR potassium channels) in glucose-stimulated insulin and glucagon secretion. Glucose metabolism in the pancreas is mainly rate limited by glucokinase [211]. Transport by GLUT2 is also associated with extra-pancreatic glucose sensing in neurones, and in blood vessels of the portal and systemic circulations. In the some brain regions, including the hypothalamus, GLUT2 and hexokinase may co-ordinately control aspects of feeding and energy expenditure [211]. Consistent with this, GLUT2 knockout mice have abnormal feeding behaviour [87] and mutations in the human *SLC2A2* gene are associated with a preference for sugar feeding and type 2 diabetes [69]. Human mutations result in the Fanconi-Bickel syndrome, originally described as a glycogen storage disorder and associated with a range of metabolic traits including glucosuria. A large range of rare inherited mutations in the *SLC2A2* gene occur. The range of phenotypic changes associated with different GLUT2 mutations include those due to a loss of GLUT2-mediated glucose transport and GLUT2 targeting to the plasma membrane [68]

### GLUT3 and GLUT14

GLUT3 is mainly present in the brain [139]. The *K*_m_ of 1–2 mM is low in relation to blood glucose and the transport catalysis rate is very fast in cerebellar granule neurones [138]. These kinetic factors allow the neurones to avidly remove and metabolise glucose that has passed through the blood-brain barrier endothelial cells [192]. Some brain cancers, such as those arising in glioblastoma cells, could be specifically targeted by therapeutic reagents because of their dependence on the high rates of GLUT3-mediated glucose uptake [45].

GLUT3 is important for mouse embryo development and (together with GLUT1) with the supply of glucose to the foetus [77, 192]. GLUT3 is upregulated in response to hypoxia [2], and this upregulation is mediated by the transcription factor HIF, possibly in response to a long non-coding RNA [128]. Depletion of cellular ATP levels also leads to upregulation of GLUT3 by a slowing of degradation [118].

GLUT14 is a GLUT3 variant that has also been found in the genome as a duplicon of GLUT3 [226]. It is essentially uncharacterised in terms of substrate specificity, which is assumed to be similar to that of GLUT3. The function and tissue distribution are uncharacterised, although there is some disease association, specifically in inflammatory bowel disease [3].

Heterozygous knockout of GLUT3 in mice leads to abnormal brain development and some behavioural abnormalities that resemble those of autistic spectrum disorders. These GLUT3 partial knockout phenotypes indicate an important role in many aspects of neuronal function [34, 182, 233].

### GLUT4

GLUT4 is present mainly in the insulin-sensitive tissues of adipose, heart and skeletal muscle. Its *K*_m_ for glucose substrate is close to the fasting blood glucose level and this is unchanged by insulin action, which instead leads to a massive increase in the *V*_max_ for transport, which can be over 10-fold in some tissues [209]. The transport system is kinetically symmetric with equal *K*_m_ and *V*_max_ for uptake, efflux and exchange transport [208].

There are three main ways in which GLUT4 can be regulated: post-translocation modification including glycosylation, possible ubiquitinoylation, phosphorylation and palmitoylation; changes in turnover including transcription and degradation; membrane protein translocation and compartmentalisation. Each of these processes would be expected to have a different degree of temporal control, ranging from rapid control of translocation to slower control of protein turnover, but it is often difficult to distinguish these separate processes. For the GLUT4 transporter, the extents of post-translational modification by ubiquitinylation [127], phosphorylation [176] and palmitoylation [64, 171] may be quite low and are not easily quantified. However, if GLUT4 traffics through low abundance but critical subcellular compartments in which these modifications occur, then these steps may be quite influential in determining the storage and fate of GLUT4 vesicles. Further studies may reveal the extent to which these modifications are important for long-term regulation of GLUT4 and control of its turnover.

The insulin-stimulated increase in the *V*_max_ for transport in insulin-target tissues occurs rapidly and is considered to be almost entirely due to the rapid translocation of GLUT4 to the cell surface from an intracellular reservoir compartment, but often it is also associated with a smaller fold translocation of GLUT1 [92], and possibly other GLUTs [145].

**Fig 1:**
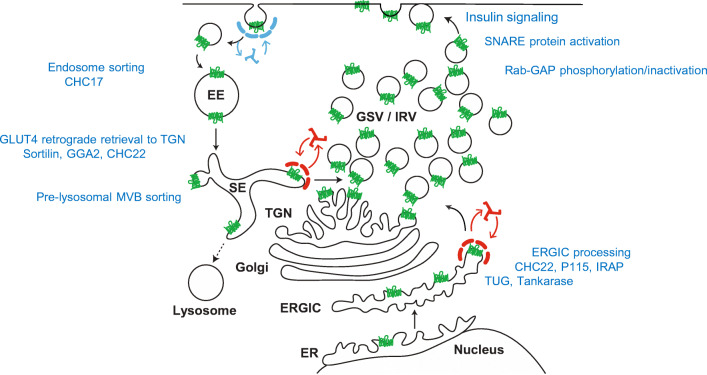
Compartmentalisation and retention of GLUT4 in insulin-target tissues. In basal cells, most of the cellular GLUT4 is sorted into intracellular compartments (EE: early endosomes; SE sorting endosomes; TGN: trans-Golgi network; Golgi: Golgi stacks; ERGIC; endoplasmic reticulum- Golgi intermediate compartment; ER endoplasmic reticulum; lysosomes: lysosomes including multi-vesicular body (MVB) pre-lysosomal compartments; GSV/IRV GLUT4 storage vesicles/insulin-responsive vesicles. Several processing and sorting steps (blue text) are involved in maintained intracellular GLUT4. These include EE retrieval of GLUT4 from the plasma membrane via CHC17 clathrin-coated vesicles. The SE compartments segregate GLUT4 either for recycling or for lysosomal degradation via MBV compartments that lead to lysosomes (this appears to be dependent on ubiquitinoylation of GLUT4 or GLUT4 associated proteins). In the absence of ubiquitinoylation, or following deubiquitinylation by USP25, vesicle recoating with clathrin (CHC22 in humans) occurs in a process involving sortilin and GGA2 and with retrograde transfer to the TGN and GSV/IRV. GLUT4 is also trafficked from ER to ERGIC. It is retained in ERGIC but is available for release (possibly directly to GSV/IRV). The candidate proteins for this further processing and sorting are clathrin-coating CHC22 in humans, the Golgi tether p115, the insulin-responsive amino-peptidase IRAP, TUG and the enzyme tankarase, UBC9 (reviewed in detail in ref [26, 120]). Insulin signalling through tyrosine phosphorylation leads to activation of SNARE proteins (VAMP2 on GLUT4 vesicles and the SNAP23, Syntaxin4, Munc18c complex at the plasma membrane). These activations facilitate fusion of GLUT4 vesicles with the plasma membrane. Insulin signalling through serine kinase cascades leads to phosphorylation and inactivation of the Rab-GAP activities of TBC1D1 and TBC1D4. This is associated with GTP loading of the Rabs on GLUT4 vesicles (mainly Rab10-GTP) and these GTP-Rabs direct the recruitment of the vesicles to the plasma membrane, ref [120] for review. This figure is adapted from the GLUT4 traffic map described in [32] and kindly supplied by the Brodsky group https://www.ucl.ac.uk/research/domains/food-metabolism-and-society/events/glut4-traffic-map-workshop

GLUT translocation was originally discovered in adipose cells in 1980 [50, 202]. This was followed by the cloning of the GLUT4 isoform [20, 101]. Since then, many new techniques for studying the subcellular locations of GLUT4 have been developed and important aspects of the link between GLUT4 traffic and insulin signalling have been revealed (reviewed in [120] and summarised in Fig. 1) Early insulin signalling from the insulin receptor tyrosine kinase activation to the downstream serine kinase Akt is now well understood [206]. Activated Akt leads to phosphorylation and inactivation of several GAP proteins that control metabolism. These include the Rab GAPs TBC1D1 and TBC1D4 which maintain their Rab substrates in the GDP form. Rab-GAP inactivation therefore leads to increases in the loading of Rab proteins with GTP. One of the main targets of TBC1D1 and TBC1D4 is Rab10, but there are possibly other Rab proteins that are involved in the control of GLUT4 traffic that operate in parallel with Rab10 or downstream of it [123, 201]. A cascade of linked Rab GAPs and Rabs is also likely [172]. TBC1D1 and TBC1D4 are also targets of AMPK and there may be some convergence with signalling from insulin activation of Akt [71, 103]. However, it has yet to be demonstrated that GLUT4 exocytosis occurs in response to AMPK signalling to the Rab GAPS. The activities of the Rab GAPs are further discussed by Al Hasani and Chadt in this Special Issue of Pflügers Archives.

GLUT4 is compartmentalised in multiple subcellular compartments in insulin-target cells [28, 32, 93, 193] and much effort has been involved in determining how and when the insulin-stimulated redistribution of GLUT4 between the plasma membrane, endosomes, pre-lysosomal multivesicular body (MBV) compartments, trans-Golgi stacks and ER-Golgi intermediate compartment (ERGIC) compartments occurs (Fig. 1) (reviewed in [25, 120]). An intracellular targeting sequences, FQQI at the N-terminus and dileucine and an acidic cluster motif at the C-terminus [18, 23, 142, 188] determine the intracellular distribution and are necessary for GLUT4 sequestration to a specialised reservoir compartment in unstimulated cells.

Kinetic studies on GLUT4 traffic have revealed a major effect of insulin is to increase the rate of exocytosis of GLUT4 from the intracellular reservoir compartment to the plasma membrane in adipose cells [180, 232] and to the sarcolemma and T-tubules in muscle [72, 113]. This change in the rate of exocytosis rate quantitatively and temporally accounts for increase in cell surface GLUT4. Several insulin-regulated steps may be involved in stimulation of GLUT4 exocytosis. These include release of GLUT4 from intracellular retention [155] and insulin-stimulated fusion of GLUT4 vesicles at the plasma membrane [9, 122]. The fusion step may be controlled directly at the plasma membrane as insulin-activated plasma membrane alone can activate fusion in a cell free system in which vesicles and cytoplasm are maintained in a basal state [122]. In addition to steps downstream of Akt activation [9], insulin-activated receptor tyrosine kinase stimulates vesicle fusion proteins of the SNARE complex through changes in syntaxin4 and Munc18c interaction [6, 104, 119, 168].

Intracellular retention of GLUT4 in a specialised reservoir compartment is essential for maintaining a low rate of glucose transport in the basal state. Retention and GLUT4 sorting mechanisms vary depending on the cell type and cell line. However, collectively the retention mechanisms appear to involve a series of interacting, adapter and tethering proteins including TUG, sortilin, golgi associated, gamma adaptin ear containing, ARF-binding protein 2 (GGA2), p115 and insulin-responsive aminopeptidase (IRAP), the clathrin heavy chain (CHC) components CHC17 and CHC22 [26, 32, 177]. The enzyme tankarase, UBC9 (ubiquitinylation) and USP25 (deubiquitnylation) also appear to be involved in post-translational control of GLUT4 traffic and sorting [124, 127, 177, 227]. Figure 1 summarises the compartments in which GLUT4 is localised, together with the GLUT4-vesicle-interacting proteins which control GLUT4 retention and sorting. These processes ultimately lead to the generation of the GLUT4 storage compartment and insulin-responsive vesicles.

In rat adipose cells, a large proportion to the total internal GLUT4 resides in specialised vesicles of approximately 60 nm that contain very few additional components other than IRAP and VAMP2 [109, 110, 169], and it is these vesicles that are rapidly recruited to the plasma membrane in response to insulin signalling. The fusion of these vesicles with the plasma membrane occurs at a rate that is consistent with this step limiting the whole GLUT4 exocytosis process [9, 122]. It would be useful to obtain rates for the insulin-stimulated release of GLUT4 from retention compartments and restraining steps, such as TUG proteolytic cleavage and changes in ubiquitylation of GLUT4 vesicle-associated proteins. This would allow determination of whether these release steps occur at rates that are comparable with the insulin-stimulated GLUT4-vesicle fusion rate.

Endocytosis of GLUT4 occurs through both clathrin-coated [70, 72, 197] and non-clathrin vesicles [22]. Although most of the insulin-stimulated translocation of GLUT4 occurs through a stimulation of exocytosis, hypoxia and cell-energy depletion (through mitochondrial electron transport inhibition) lead mainly to reductions in GLUT4 endocytosis. This is evident from experiments in which metformin, hypoxia, oligomycin and DNP treatments have been studied [4, 165, 229, 230]. Exercise and insulin action impinge on GLUT4 traffic through different mechanisms [71, 173]. These effects may be partly related to changes in the AMP/ATP level and activation of AMPK [4, 113, 121, 125]. Stimulation of the levels of cell surface GLUT4 through inhibition of endocytosis is necessarily much slower than that mediated by stimulation of exocytosis [93, 113]. Many of the steps involved in synthesis, degradation and post-translational modification of GLUT4 might also be expected to be slow processes. However, GLUT4 endocytosis at the plasma membrane, and steps that lead to GLUT4 degradation through pre-lysosomal compartments, may be drug targets for treatment of type 2 diabetes as they may bypass defects in insulin signalling to GLUT4 exocytosis. Long-term regulation and dysregulation of these processes may occur. Exercise and training lead to an upregulation of GLUT4 in skeletal muscle [90], while in severe type 2 diabetes GLUT4, and the ubiquitin conjugating enzyme UBC9 are both lost in both adipose tissue and muscle [108], but the importance of these correlations is unclear.

Homozygous knockout of GLUT4 in mice is not lethal, while heterozygous GLUT4 knockout mice develop diabetic histo-pathologies but are lean [114, 147]. Transgenic mice in which GLUT4 is over-expressed in muscle have improved glucose disposal [218].

## Class 2: GLUTs 5, 7, 9 and 11

One of the distinguishing features of class 2 GLUTs is the occurrence of the exofacial glycation site on the arginine in exofacial loop 5 (the linker between TM9 and TM10), with no glycation site on loop1 as in class 1 and class 3 GLUTs. There seems to be preference for fructose rather than glucose as substrate. There are alternative non-hexose substrates for some class 2 transporters.

### GLUT5

GLUT5 was cloned and sequenced from the small intestine [116]. Levels of GLUT5 are regulated in response to changes in the levels of fructose in the intestine [47, 100]. GLUT5 is a fructose transporter with higher affinity for fructose than glucose [29, 207] and is abundant in the small intestine, kidney and sperm with lower levels in fat and skeletal muscle [62].

In adipose and muscle, GLUT5-mediated fructose transport is responsive to insulin stimulation [98]. GLUT5 is upregulated in the skeletal muscle and intestine in type 2 diabetes patients and reversed by treatment with anti-diabetes drugs [198]. Recently there has been a major advance in the study of GLUT5 with the determination of the 3D crystal structures of GLUT5 by the Drew group [158].

### GLUT7

GLUT7 was found at high levels in the small intestine, colon, testis and prostate [131]. The GLUT7 substrate remains obscure and controversial. Manolescu et al. [140, 141] studied GLUT7 in Xenopus oocytes and reported that GLUT7 was a high affinity and low capacity (*V*_max_) fructose transporter. However, more recent studies have shed some doubt on whether fructose is a substrate for GLUT7. Ebert et al. [65, 66] have shown that in comparison with GLUT5, GLUT7 has negligible fructose transport in mammalian NIH-3T3 fibroblasts. Differences between oocyte and mammalian cell expression data may be related to a leak pathway and assay conditions that lead to trapping of fructose by a low *K*_m_ fructokinase. The comparison between GLUT5 and GLUT7 is therefore useful and it has been observed that GLUT7 domains introduced into GLUT5 generate functionally inactive chimeras [65].

### GLUT9

GLUT9 was identified by the Moley group [164]. It has two splice variants and is present at highest levels in the liver and kidney. The splice variations have different tissue distributions and subcellular localisations. GLUT9 is important in early development and the pre-implanted embryo [7]. GLUT9 was originally proposed to transport fructose based on studies in Xenopus oocytes [141]. Later studies, also working with the Xenopus expression system, found insignificant levels of fructose transport and instead reported that GLUT9 was a urate transporter [5, 40, 194]. Knockout of GLUT9 in mice led to hyperuricemia, hyperuricosuria and metabolic syndrome [54], consistent with a major function for this transporter in urate transport. Although urate seems to be the most important substrate, exchange of urate for glucose and fructose in the kidney has been reported suggesting that urate loss may be usefully accompanied by sugar reabsorption [37].

### GLUT11

Cloning and characterisation of GLUT11 revealed that it transports both glucose and fructose [59, 179]. Its sequence has highest similarity to GLUT5, and this suggests the possibility that fructose may be the preferred substrate. GLUT11 is absent in mice and so it is likely that GLUT11 function is non-essential. It is expressed mainly in the skeletal muscle and heart. In the human skeletal muscle, GLUT11 has a fibre-type specific distribution and is abundant in slow-twitch but not fast twitch fibres [78]. Three different length transcripts occur. All of these transcripts can transport fructose but they each have somewhat different tissue distributions [181].

## Class 3: GLUTs 6, 8, 10, 12 and GLUT13 (HTMI)

This class of protein has the typical 12 TM spans. The asparagine N-linked glycation to asparagine occurs in loop 1. This class of GLUTs has intracellular targeting sequences, typically dileucine motifs, within the N-terminal domains. The presence of these sequences suggests at least some intracellular targeting and compartmentalisation that may be related to function.

### GLUT6

GLUT6 was identified and cloned by the Joost group [58], but initially named GLUT9. The preferred substrate for GLUT6 is unclear but it has low affinity for glucose and fructose [107]. It is present mainly in the spleen, lymphocytes and brain. GLUT6 is upregulated in inflammatory cells [35, 115, 137] and endothelial cells [212]. GLUT6 has an N-terminal dileucine group that is associated with intracellular retention [132]. It may be localised primarily to lysosomal membranes [137]. GLUT6 knockout has minimal effects on glucose metabolic physiology in mice [31].

### GLUT8

GLUT8 was identified by the Joost and Mueckler groups [33, 60]. GLUT8 contains a lysosomal targeting motif and localises to late endosomes and lysosomes [8, 184, 225]. It transports glucose and fructose and is upregulated (along with GLUT12) on high-fat diets [53].

GLUT8 has recently been demonstrated to transport the disaccharide trehalose, and it has homology to the trehalose transport Tret1 in drosophila [143]. It appears to be present at low levels in many tissues but is high in testis [60], spermatozoa [83] and in lactating mammary gland alveolar cells [217]. In the latter cell type, it plays a role in lactose synthesis and accumulation [217]. It is important in the blastocyst and in these cells, it translocates to the plasma membrane in response to insulin. In the early stages of development, it may functionally act instead of GLUT4 (which is absent) [33].

As trehalose and lactose are GLUT8 substrates, it would be of interest to explore the range of disaccharides that GLUT8 transports and further explore whether its subcellular translocation is responsive to hormonal stimulation. GLUT8 knockout mice have a mild phenotype with evidence for reduced sperm motility and some abnormal neuronal and locomotor activities [183].

### GLUT10

GLUT10 is somewhat larger than most GLUTs with 541 amino acids. It occurs at high levels in the liver and endocrine pancreas [51, 146]. It is also present in vascular smooth muscle cells where it has mainly an intracellular distribution [166]. Human GLUT10 mutations are associated with tortuosity and stenosis of large arteries leading to a propensity for aneurysms [24, 46]. In artery cells, there is intracellular targeting of GLUT10 to mitochondria, where it functions in redox control through the accumulation of dehydro-ascorbate [24]. Consistent with a role of GLUT10 in compartmentalisation of ascorbate within arterial cells, mice with a GLUT10 G128E mutation exhibit increased levels of reactive oxygen species, fragmented mitochondria and cross-linking of proteins at the endoplasmic reticulum [203].

### GLUT12

GLUT12 is another large protein of 596 amino acids. It is mainly present in insulin-responsive, adipose, heart and skeletal muscle cells and mammary gland alveolar cells [136, 199]. In unstimulated cells, it localises to intracellular vesicle associated with the Golgi and at the plasma membrane [1, 74] in a manner that is dependent on its dileucine targeting motif. It is recruited to the plasma membrane of its target tissues in response to insulin stimulation [199]. It is important in foetal development, particularly of the heart, while GLUT12 deficiency is associated with heart failure [105]. GLUT12 expression is upregulated by androgens in the prostate and levels are increased in prostate cancers [10, 39, 224].

### GLUT13

GLUT13 is a very large protein of 618 amino acids and has clear specificity for myo-inositol in a manner that is facilitated by protons. It is therefore more commonly named HMIT or H(+)-myo-inositol transporter [213]. It is mainly localised to neuronal tissues [57]. Its activity can be regulated by changes in cell membrane potential but its coupling to proton movement may not be obligatory.

## Crystal structure of GLUTs

In 2014, Yan and colleagues described the crystal structure of GLUT1 with bound to nonyl-β-D-glucoside [56]. This structure has in an inward-facing conformation. The same GLUT1 structural conformation was crystallised with the GLUT inhibitor cytochalasin B bound [111]. This was followed in 2015 by the reporting of a crystal structure of GLUT3 in outward-facing conformations. Structures with GLUT3 bound to glucose or to the disaccharide maltose were obtained [55].

**Fig. 2:**
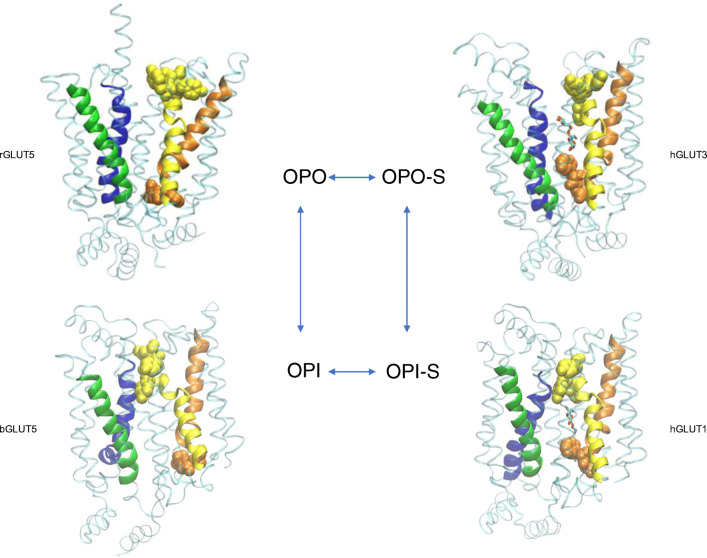
Crystal structures of GLUT proteins define the structural basis for alternate exposure of binding site clefts to either outside or inside solutions. Discrete conformations for the GLUTs are identified as OPO (open-outside without substrate; rat GLUT5, pdb:4ybq), OPO-S (open-outside with substrate; human GLUT3 with maltose, pdb:4zwc), OPI-S (open-inside with substrate; human GLUT1 with nonyl-glucoside, 4pyp) and OPI (open-inside without substrate; bovine GLUT5, pdb:4yb9). The structural fold has four inverted trimer repeats with TM1-3 and TM7-9 showing inverted repeat similarity to TM4-6 and TM10-12, respectively. The first TM helix of each of the four trimers is shown as cartoon representation: TM1 (blue); TM4 (green); TM7 (yellow); TM10 (orange), while the rest of the protein is shown as transparent ribbon. The upper half of TM7 (TM7b) and the lower half of TM10 (TM10b) are particularly important for occluding the binding site from the external and internal solutions, respectively. The occlusion OPO to OPI is associated with hydrophobic residues in TM7b moving closer to TM1 while hydrophobic residues in TM10b move away from TM4. The reverse occurs in the OPI to OPO conformational changes. TM7b hydrophobic residues 291, 292, 293 and 294 and TM10b residues 386 and 387 in GLUT1 (with equivalent residues in GLUT3 and GLUT5) are shown with space filling to illustrate this occlusion. In addition, salt bridges between residues at the cytosolic ends of the TMs are formed to bunch these TM ends, and the C-terminal ICH5 region, closer together in the OPO conformations. The location of the substrate glucose moiety that is revealed from both the GLUT3 structures with maltose (OPO-S, and GLUT1 with nonyl-glucose (OPI-S) is the same as in the GLUT3 structure with glucose (not shown, pdb:4zw9). In all cases, the glucose in the central site is polarised with C1-O projecting toward the internal solution with C4-O trailing. The structures shown are constructed using the VMD software and using the pdb files reported and described by the Yan group ([55, 56] for GLUT1, 3) and the Drew group ([158] for GLUT5)

The structure of GLUT1 with nonyl-β-D-glucoside bound to the central substrate site revealed a cleft which is open to the inside solution of the cell, with the cleft to the outside clearly occluded and inaccessible (Fig. 2 OPI-S). By contrast, the structure of GLUT3 with exofacially bound maltose has a cleft only open to the outside solution. The leading glucose moiety occupies a deep central region of the cleft while the trailing glucose moiety of the disaccharide (the non-reducing end) lies nearer the surface of the cleft but is still well within the cleft (Fig. 2 OPO-S). In a separate crystal structure with bound glucose in GLUT3 (pdb:4zw9), the sugar occupies the same central location and orientation as that which occurs in the maltose and nonyl-β-D-glucoside structures. In each case, the C4/C6-OH of the glucose trails behind the leading C1-OH. The central position of the bound glucose moiety remains essentially the same in all three structures while the protein changes shape around the ligand in the OPO-S to OPI-S transition.

Crystal structures of GLUT5 have been obtained by the Drew group [158]. GLUT5 has been crystallised (without the substrate fructose present) but in both outward open (rat GLUT5, OPO) and inward open (bovine GLUT5, OPI) conformations. All 4 structures, OPO; open-outside: OPI; open-outside with substrate bound: OPO-S; open-inside with substrate bound: OPI-S, show the same basic protein structural folds. Having all 4 structures strongly supports the alternating access hypothesis for transport in which there are cyclic transitions between the 4 conformations OPO→ OPO-S→ OPI-S→ OPI and back to OPO. The mechanism of alternate binding-cleft opening based on descriptions of the GLUT1, GLUT3 and GLUT5 structures [55, 56, 158, 200] and homologous bacterial glucose transporters [200] are remarkably consistent. There is a pseudo 2-fold axis of symmetry such that the locations of helices in the C- and N-terminal halves are superimposable. In addition, within each half of the proteins (6 helices), there is trimer symmetry such that trimer TM1,2,3 has inverted repeat sequence similarities to trimer TM4,5,6 while trimer TM7,8,9 has inverted repeat sequence similarities to trimer TM10,11,12. This enables 4 trimer substructures to move relative to each other. The OPO to OPI transitions are partly due to rigid-body rotation and tilt of the helices in the four trimer sections of the protein. The N-terminal half may act as a platform for the conformational changes and hydrogen bonding interactions with substrate that occur in the C-terminal half [55, 158].

The outward-facing conformations are stabilised by salt bridge formation between conserved glutamates and arginines of endofacial loop sequences RXGRR that occurs between TM3-TM4 and TM10-TM11. It is these stabilising motifs that are typical of the whole of the *SLC* family of transporters extending beyond the *SLC2* family of glucose transporters. Previous mutagenesis studies highlighted the importance of these interactions in stabilising the outward-facing conformation. Mutation of conserved glutamates and arginines leads to a locking of GLUT4 into the inward-facing conformation that could not bind exofacial ligand Bio-LC-ATB-BMPA but could bind the endofacial ligand cytochalasin B [186]. Furthermore, a GLUT1 mutation in conserved glutamate 329 (E329Q) facilitated crystallisation of GLUT1 in the inward-facing formation [56]. In the inward-facing conformations, salt bridges between these helical segments are lost and instead there is salt bridging to the cytoplasmic loop domains between TM6 and TM7 (ICH1-4) and the C-terminus ICH5. The C-terminal segment ICH5 moves to open an ICH network of interactions, and this may be associated with more access to the binding site from the internal side (Fig. 2).

In addition to the rigid body movement, the crystallographic studies on GLUT1, GLUT3,GLUT5 and the bacterial homologue XylE now highlight the importance of localised movement of helical segments and side chains within the C-terminal half of the protein, and particularly with movement of the top half of TM7 (TM7b) and its inverted repeat of TM10 (TM10b) [55, 158]. These half-helices occur at hinge points that contain multiple glycine residues [204]. Thus, TM7 and TM10 both have broken helical shapes so that when they move, the substrate-binding cavity opens to either the outside or the inside. The TM7B sequence G_286_INAVFYYST_295_ is highly important in sugar binding and transport as revealed both in mutagenesis studies [61, 96, 160, 220]. Furthermore, a threonine 295 mutation to methionine has been found to be a human GLUT1 haploinsufficiency disease [222]. In particular, the sequence FYYST is of interest as mutations in these residues produce a stable outward-facing conformation that binds the exofacial reagent Bio-LC-ATB-BMPA but not the endofacial reagent cytochalasin B and very low glucose transport activity. Therefore, it appears that the entire TM7B sequence may have a role in opening and closing access (occluding) at the exofacial side of the GLUT transporters. Comparisons of crystal structures of GLUT1, GLUT3 and GLUT5, and illustrated in Fig. 2, reveal that movements of hydrophobic side chain groups of tyrosine, tryptophan and phenylalanine residues are involved in shutting off the glucose cleft from the external solution (involving TM7b residues Phe291, Tyr292 and Tyr293 in GLUT1 and the equivalent Tyr, Tyr, Tyr in GLUT5). Mutagenesis studies highlighted the importance of these hydrophobic regions in TM7b [61, 96, 150, 160, 220]. The same conformational change occurs both with and without substrate (Fig. 2). Residues in TM7b close against residues in TM1 to occlude the outside site while residues in TM10b move away from TM4 to open the inside site. When the reverse occurs, with TM7b moving away from TM1 and TM10b moving toward TM4, then the outside site opens and the internal site closes (Fig. 2).

The availability of crystal structures for the GLUTs is a tremendous advance and will pave the way for more studies that reveal the dynamics of the transport catalysis process and provide information that will be useful in the design of GLUT-specific inhibitors. The crystallography data strongly support the alternating access model for transport that was proposed for the phosphate exchanger by Mitchell [148] and for the Na/K ATPase by Jardetzky [102]. Experimental evidence for the applicability of this hypothesis to GLUT proteins was first obtained from analogue probes specific for either OPO-S or OPI-S conformations [14, 94] (section: GLUT specificity for substrates and inhibitors).

## Inferred transport catalysis mechanism

The alternative access mechanism is distinct from a mechanism in which there is free movement of glucose through an ungated membrane pore or channel. The distinguishing characteristics of glucose transport that must be accounted for mechanistically are the related phenomena of substrate exchange and counterflow [196]. Counterflow can best be described by considering two substrates (although it is often measured by following the fluxes of radioactive tracers of a single substrate). When substrate A in the external solution is exchanged with substrate B in the internal solution, then it can be temporarily driven up its concentration gradient (so that A-inside > A-outside) while B is moving down its concentration gradient. Experimentally, counterflow has proven to be very useful in assays as the transport signal from A is enhanced [55]. Counterflow stops only when both A and B are at equilibrium concentrations. A simple pore model cannot account for this phenomenon. B moving down its concentration gradient through a simple pore might be expected to inhibit the flux of A, but this is not observed. The GLUTs are not trans-inhibited by substrate on the opposing side of the membrane [81]. For GLUT1 in erythrocytes, trans-acceleration is observed such that B increases the initial rate of transport of A. The alternate access model accounts for exchange and counterflow as the single central site is not exposed to both external and internal solutions at the same time, and therefore A and B do not compete. It can also account for accelerated exchange. If the conformational change OPI-S→ OPO-S is faster than OPI→ OPO (Fig. 2), then exchange flux can be faster than net influx which would be rate limited only by OPI→ OPO. The GLUT1 crystallography data indicate a very restrictive and narrow choke point within the transporter (around Trp288), Fig. 2 in OPI-S. This provides strong evidence that sugars A and B, initially from opposite sides of the membrane, would not be able to move past each other at this point. In addition, sugar analogue studies (section: GLUT specificity for substrates and inhibitors) suggest that the sugar is tightly polarised in its orientation within this central region. A comparative molecular dynamic study on the plant SWEET and bacterial semi-SWEET glucose transporters has suggested a sliding movement of glucose (with restricted orientation) in SWEET transporters while semi-SWEET transporters have a tumbling movement (with a less restricted orientation of glucose). The sliding movement was associated with lower free energy and faster transport, possibly associated with less steric clashes with the protein during the transport [41].

**Fig. 3:**
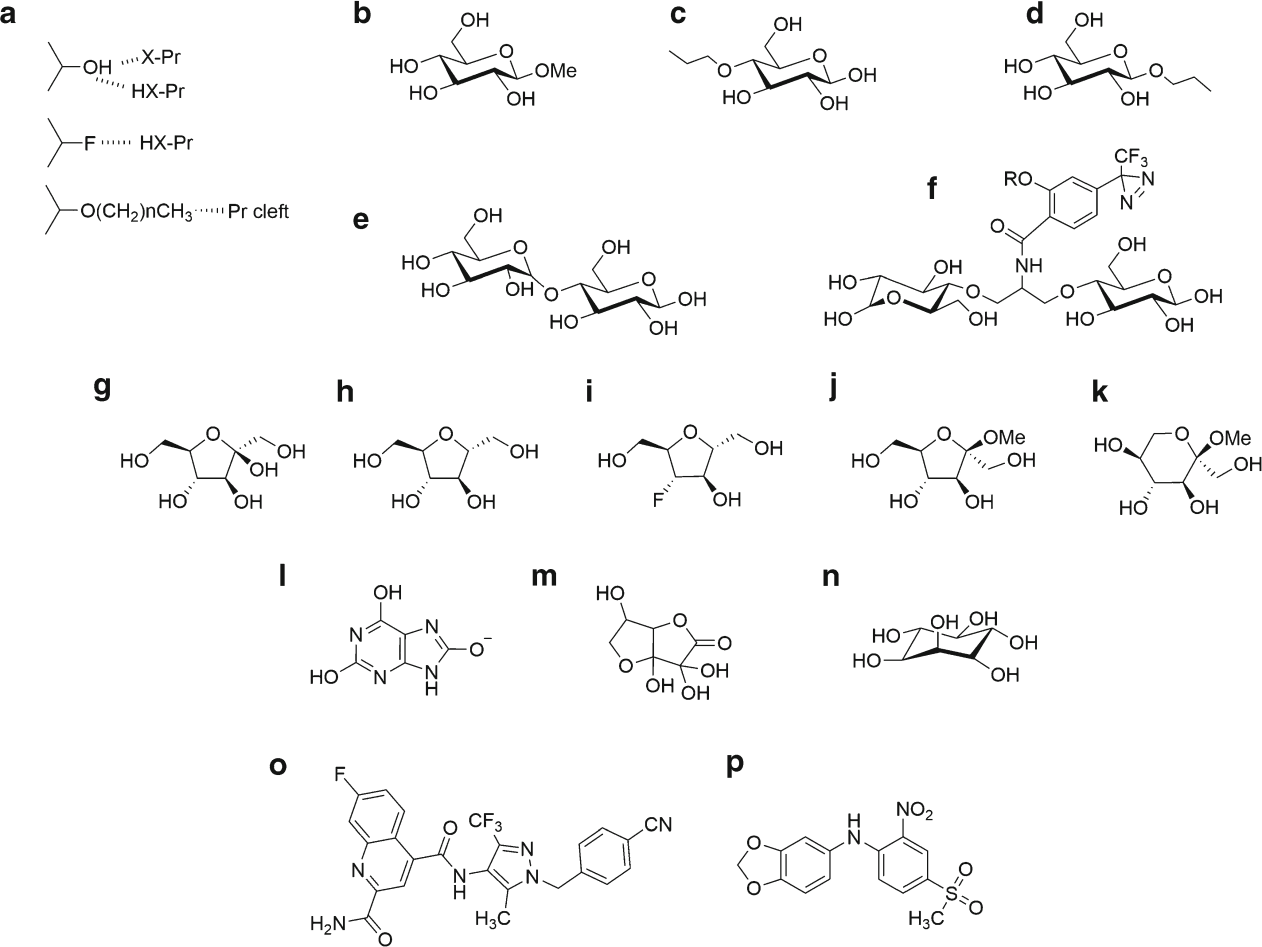
Substrates and inhibitors reveal differing specificity requirements for the GLUTs and may aid the therapeutic targeting of individual GLUTs that are implicated in disease. In **a** substrate and inhibitor, interactions with the GLUTs are associated with H-bonding (involving either electron donating or withdrawing groups) that can be examined using analogues that are H-bond accepting only (fluorine substitution for –OH). Spatial limitations to binding can be explored using O-alkyl groups. In **b,** β-methyl-D-glucoside has very low affinity for the outside site of GLUT1 suggesting a close approach to C1-O, while a 4-O-propyl group (**c)** is well tolerated at the outside site. The outside site can accommodate quite bulky substitutions at C4-OH with disaccharides such as maltose (**e** and Fig. 2 in OPO-S), and bis-glucose propylamine BGPA (**f**) derivatives being well tolerated**.** In **f**, the R group on the phenyl-diazirine photoreactive moiety can be a very large spacer arm with biotin. In contrast to these spatial restraints at the outside site, C1-O substitutions as in β-O-propyl-glucoside (**d)** or β-O-nonyl-glucoside (Fig. 2 in OPI-S) are well tolerated at the inward-facing site. Both fructofuranose and fructopyranose forms of fructose are transported by GLUTs such as GLUT5. The closed ring forms, including 2-5-anhydro-D-mannitol (**h**) and the β-methyl-fructofuranosides and β-methyl-fructopyranosides (**j** and **k**), are good substrates and inhibitors. Several new derivatives, including fluorescent and photolabeling compounds, based on 2-5-anhydro-D-mannitol have been described. The introduction of an H-bond accepting fluoro group at C3 of 2-5-anhydro-D-mannitol (**i**) reduces affinity for GLUT5 but increases affinity for GLUT1 suggesting tuning analogues for a specific GLUT is possible. The GLUT family is not restricted to glucose or fructose as substrate. GLUT9 is a transporter for urate (**l**). Dehydro-ascorbate in solution as a hydrate (**m**) is transported well by several GLUTs, and particularly GLUT10. GLUT13 transports myo-inositol (**n**) with good specificity. Now that GLUT structures are available, therapeutic targeting of individual GLUTs is becoming possible. In silico docking-aided screening of compound libraries has led to the identification of Bay 876 (**o**) as a high affinity inhibitor of GLUT1 (and not other class 1 GLUTs) and MSNBA (**p**) as a specific inhibitor of GLUT5 with negligible affinity for class 1 GLUTs

More complex multiple-occupancy pore models for glucose transport have been proposed in which sugars A and B move past each other within the protein and do not impede each other’s progress [48]. Currently, it is not clear whether the central polarisation of substrate in the GLUT crystal structures is maintained in the wide clefts leading to the central binding site, but less restricted orientation within the wider regions of the cleft is likely. The crystallography data indicate that the cavities leading to the binding site are certainly large enough at the exofacial site to accommodate the trailing glucose of maltose and bis-hexoses (see Fig. 2 OPO-S and Fig. 3 e and f). Cis-allostery in which multiple occupancy by substrate and/or inhibitor occurs at the exofacial site of GLUT1 has been demonstrated [159]. However, cis-allostery is consistent with the alternating access mechanism [133]. Trans-allostery (with the possibility that substrates and inhibitors from opposite sides of the membrane bind simultaneously and bypass one another within the protein) is a much more controversial issue [134, 154, 156, 157]. Trans-allostery would be consistent with the alternating access mechanism if the simultaneous trans-occupancy occurred within a dimer of two alternating-access monomers that were conformationally linked. One monomer could be in the OPO-S while the other was in the OPI-S conformation [52, 134]. Although much of the literature suggests that GLUTs behaves as monomers in most situations [154], it is possible that when GLUT1 levels are very high within cells (such as human erythrocytes), then dimerization and associated allosteric-like effects are more likely.

## GLUT specificity for substrates, inhibitors and drug targets

Defining the interactions of GLUTs with substrates is often useful in attributing the function of the GLUT. Most GLUTs exhibit some level of glucose or hexose transport. However, it is now clear that although they have the same structural folds, which are associated with similar modes of transport catalysis mechanism, often quite subtle differences in amino acid side chains can radically alter substrate preference and interaction. Likewise, subtle differences in substrate analogues can alter which of the GLUTs transports the analogue most efficiently. Considerations of GLUT specificity are therefore ongoing and much needs to be done in terms of defining both the physiological substrates and the related functions of some of the class 2 and class 3 transporters.

### GLUT specificity for substrates and inhibitors

The specificity and transport kinetic features of the GLUT proteins for hexose analogues and transport inhibitors have been most extensively studied for class 1 (GLUTs1-4) and for GLUT5. Such studies are important for defining the key features of the substrate that are necessary for efficient transport, for defining differences between each of the GLUT proteins and ultimately for defining routes to the development of high affinity transport inhibitors

Sugar analogues including deoxy sugars and epimers have been useful in defining these substrate specificities in GLUTs1-4 [14, 43, 170]. The hydroxyls at C1, C3, C4 and C6 and the ring oxygen are all important for hexose binding to GLUT1. The C4 hydroxyl seems to be more important for binding to GLUT1 where galactose has 10-fold lower affinity than glucose. By contrast, GLUT4 and GLUT3 have similar affinity for glucose and galactose [43, 170]. The C2 hydroxy seems to be unimportant for transport as 2-deoxy-glucose is transported well by all class 1 GLUTs. The direction of H-bonding from glucose hydroxyls to both GLUT1, GLUT3 and GLUT4 transporters has been examined using fluorine substitution. If the bond is from the protein sidechain to the electronegative O of the –OH, then the electronegative F will substitute. If the H-bond involves this H of the –OH, then it will not. Fluorine substituted D-glucoses indicate H-bonding to the C1-β-O of glucose and to the C3-O, C4-OH, C6-O positions [14], Fig. 3a.

Many GLUT protein residues interact with glucose in the deep binding cleft identified by the GLUT1 and GLUT3 crystallography studies [55, 56]. These include polar residues Gln282, Gln283, Asn288, Asn317 and Glu380 in the C-terminal half of the protein (GLUT1 numbering). Only Gln161 in the N-terminal half of the protein contributes to the H-bonding network. However, these H-bonding interactions are not all essential as single amino acid mutations do not always lead to a large loss of function [88, 96]. This probably occurs in the single point mutation variants because there is sufficient H-bonding to the substrate from other residues that surround the glucose, and/or assist its passage to and from the central binding site.

Glucose has α- and β-glucopyranose anomers in solution (in the ratio 66% β, and 33% α) and vanishing small amounts of glucofuranose forms. Studies on C1-fluoro glucoses (which do not interconvert) suggest that the β anomer binds more strongly than the α isomer [14] and is transported more rapidly by GLUT1 [135]. However, additional experiments in which glucose anomer mixtures are studied [130, 189] suggest that the α anomer is preferred. These experiments are complicated by rapid interconversion of α and β glucose anomers and by hetero exchanges (αβ and βα, exchanges). The latter dominates the transport kinetics and particularly the maximum rate of transport, which is the sum of the maximum exchange rates for α + β, no matter which anomer is being followed [170]. However, the rate of transport at low substrate concentrations (or the ratio of apparent *K*_m_ over apparent *V*_max_) is clearly faster for the α anomer [189]. Furthermore, in the crystal structure of GLUT3, both α and β glucose are present but α glucose is more abundant [55].

Studies on alkyl substitutions on the glucose hydroxyls have been informative and have provided experimental evidence for an alternating cleft hypothesis for the conformational changes that alter binding specificity at external and internal sites (section: Crystal structure of GLUTs). C1-substituted β-O-methyl glucose is not an inhibitor of substrate transport when added to the outside of the erythrocyte GLUT1 or adipocyte GLUT4. This suggests that the relatively small methyl group is not tolerated at the exofacial site of GLUT1 and GLUT4 and that there is a close approach between the C1-OH position of glucose and the protein cleft. By contrast, there is good tolerance of bulky substitutions around C4 and C6 at the external site of GLUT1; for example, 4-O-propyl-D-glucose, 6-O-propyl-D-galactose, 4,6-O-ethylidene-D-glucose and maltose bind well from outside the cell. However, these analogues are not transported by the GLUTs suggesting that bulk around C4-O/C6-O prevents a conformational change associated with transport. Hydrophobic substitutions at C6-O lead to increased binding of the inhibitors which is consistent with interaction of the substituted groups with hydrophobic amino acid side chains in the binding cleft [14, 151].

Binding constraints are different at the internal side of the GLUTs. C1-O-alkyl glucose compounds do not enter the cell through GLUT1 or GLUT4. However, a long chain glucoside (propyl-β-D-glucoside) (Fig. 3d) enters through the membrane lipid and is a good inhibitor from the inside site of both these transporters. The studies with alkyl substitutions at either C1-O or C4-O thus reveal a polarised binding of glucose at the centre of the protein with the substrate binding site alternately opening to either the inside or the outside solutions [13] such that these sites accommodated either substitutions at C-4-O in OPO-S or C-1-O in OPI-S. These specificity factors are now evident in the crystal structures with the C4-OH substituted maltose binding well in OPO-S and nonyl-glucoside binding well in OPI-S (Fig. 2).

Bis-hexose analogues with bulky substitutions into the C4-OH position have also been useful for development of photoaffinity labels that cross-link to the exofacial site of the GLUT proteins (Fig. 3f). The bis-hexose structure with hydrophobic substitutions in the trailing C4-OH position has affinities for the GLUTs that are 10-fold higher than the parent compound D-glucose. The bis-mannose and bis-glucose photolabels have been used extensively for tracking the kinetics of GLUT movement to and from the plasma membrane in response to insulin (reviewed in [91]).

Fructose adopts α and β forms of fructopyranose and fructofuranose in solution (αP:βP:αF:βF; < 1%:75%:4%:21%). GLUT5 mainly interacts with β forms since both ring-closed furanose and pyranose derivatives are good inhibitors of transport with *K*_i_ values comparable with the *K*_m_ of D-fructose (Fig. 3 j and k). C2-β-O-methyl D-frucofuranoside and C2-β-O-methyl-D-fructopyranoside both have approx. 5-fold higher affinity than the corresponding C2-α-O-methyl compounds [207]. 2,5-Anhydro-D-mannitol (2-deoxy-D-fructofuranose, Fig. 3h) is a particularly useful GLUT5 analogue that retains a furanose ring shape, as it cannot ring open and therefore cannot form anomers. Fructose transporter photolabels, based on the 2,5-anhydro-D-mannitol core structure, interact well with GLUT5 [228]. Fluorescent GLUT5 probes based on the 2,5 anhydro-mannitol ring have also recently been designed [17, 126]. The presence of a hydrogen bond donor at the C-3 position of 2,5-anhydro-D-mannitol derivatives is essential for effective binding to GLUT5 and fructose transport into tumour cells. Interestingly, replacement of the C3-OH with a fluorine group (Fig. 3i), that can function only as a hydrogen bond acceptor, resulted in selective recognition by GLUT1 rather than GLUT5 [126].

The crystal structure of GLUT5 has been obtained without bound substrate but inferences have been drawn concerning binding interactions with D-fructose. An important feature of GLUT5 structure that is distinct from the structure of class 1 GLUTs occurs in the binding cleft and at residue Trp388 (GLUT1 numbering). This tryptophan is absent in GLUT5 which instead has alanine at the equivalent position. This difference makes the GLUT5-binding cavity less sterically restrictive (Fig. 2 OPO and OPI). The lack of Trp388 also accounts for the lack of inhibition of fructose transport by cytochalasin B, a powerful inhibitor of the class 1 GLUTs which is known to interact with Trp388 [158]. A replacement of the GLUT5 Ala for Trp leads to reduced fructose transport and some gain of glucose transport [79]. GLUT5 and GLUT7 have many sequence similarities. However, GLUT5 is a much better transporter for fructose than GLUT7. Nomura et al. [158] identified that this difference could be accounted for by the absence of Gln166 (GLUT5) which is Glu in GLUT7. They found that a GLUT5 Q166E mutation led to much reduced fructose binding but improved glucose binding. Fructose transport activity of this mutant has also been reported to be impaired, but evidence for improved glucose transport was not obtained [65].

DHA is transported by many GLUTs but detailed comparison of the relative transport rates through each of the proteins in the 3 classes of GLUTs is lacking. However, it has been reported that GLUT1, 3 and 4 are DHA transporters but GLUT5 and GLUT2 are not [174, 216]. Whether this affinity for DHA is related to the ability to transport glucose, not fructose remains to be investigated. The function of DHA transport may lie in maintaining redox levels in cells (for example in human erythrocytes with high GLUT1 levels) or in subcellular compartments [178]. GLUT10 deficiency is associated with blood vessel damage [24, 203], while a deficiency in GLUT14 transport of DHA is associated with inflammatory bowel disease [3]. A more complete study of all the GLUTs that transport DHA is necessary before ascorbate is considered the main substrate for any of the GLUT proteins. In addition, it is necessary to establish whether circulating glucose levels competitively compete with DHA uptake. Other proteins of the *SLC* family including the ion-coupled transporters seem to be primarily associated with ascorbate transport [30]. Regardless of the unknown physiological significance of DHA transport by the GLUT proteins, it is of interest to discover whether the planar structure of this molecule (Fig. 3m) allows less steric constraint to transport than that which occurs during the transport of glucose and fructose. The transport of urate must in some way be determined by its pharmacophore of neighbouring steric and electronic features (Fig. 3l) but relating these features to the structure of hexoses that share the same transport protein is challenging. However, myo-inositol (Fig 3n) is structurally similar to glucose, and the extent of GLUT13 evolutionary adaptation of ancestral *SLC2* genes for specialised transport of this polyol would probably have been minimal.

### GLUT proteins as potential drug targets

With the availability of the new crystallographic data on the GLUT family of proteins, it is likely that new reagents and substrate analogues can be developed that are specific for individual GLUTs. It has long been clear that pharmaceutical control of glucose transport is desirable, particularly in the cancer biology field [190, 195, 205]. However, as glucose uptake is so essential to all cells of the body, a generalised knockdown of GLUT activity is unlikely to be useful. In addition, preventing glucose entering cells may in some cases be desirable but could potentially lead to hyperglycaemia which is clearly undesirable. There are now increasing numbers of chemical biology studies in the literature that use chemical library screening to identify GLUT inhibitors [205]. These are often based on cell screens used in conjunction with in silico screens that involve the docking of potential inhibitors on computer models of known crystal structures of the GLUTs

A GLUT1-specific inhibitor might be useful as this isoform is known to be upregulated in many cancers. GLUT1-mediated glucose transport is associated with the reliance of cancer cells on glycolytic metabolism, the Warburg effect [2, 223]. One of the many typical screens has been described by Siebeneicher et al. [190]. This group initially screened 3 million compounds and then took the lead compound and further chemically modified it to end up with the compound Bay 876 that has low nM affinity for GLUT1, but much less affinity for the other GLUTs tested. The Siebeneicher group refined their GLUT inhibitors by obtaining a crystal structure of GLUT1 with bound cytochalasin B. A range of highly modified peptide-like compounds (Glut-i1 and Glut-i2) that bound to the same inward-facing site as cytochalasin B were studied in detailed crystal structures with bound ligands [111]. Of course, there is no information yet on the range of off-target interactions that these screening-derived compounds might have. However, there seems to be real potential for GLUT-specific targeting and possibly GLUT-inhibitor-based therapies.

GLUT5 is known to be upregulated in cancer of some tissues [126, 205, 231] and fructose (derived from all the sucrose we consume) probably does more harm than good in the body as it can contribute toward non-alcoholic fatty liver disease [63]. In addition, fructose is more rapidly converted to advanced glycation end products than glucose, but the significance of this fructation is debated [86]. As described above, fructopyranose and fructofuranose analogues that are specific for fructose transporters have been synthesised. The potential for targeting the unique specificity of GLUT5 is therefore good [80, 126, 195], particularly now that crystal structures of GLUT5 are available [158]. Detailed modelling is required to design fructose analogues that are transported only by GLUT5 and not by GLUT2 (which has significant affinity for fructose [21, 44, 80]). The compound MSNBA (Fig 3p) was obtained from library screening and in silico docking and modelling [80]. It is a specific inhibitor for GLUT5 with negligible affinity for class 1 GLUTs. Importantly it has no affinity for GLUT2. Docking studies have suggested that the H-bond acceptor nitro moiety binds strongly to protonated His387, which is unique to GLUT5 while other GLUTs have a Phe group at the equivalent position.

GLUT4-mediated glucose transport into the major tissues of fat, muscle and heart is of vital importance and reductions in signalling to this process lead to insulin resistance. Transgenic mouse models of diabetes in which GLUT4 expression is increased in muscle have improvements in whole body glucose homeostasis [218]. Furthermore maintenance of normal blood glucose levels through insulin therapy is recognised as being important in any hospitalised patients undergoing intensive critical care [214]. Clearly insulin resistance in mammals is undesirable and inhibition of this major protein responsible for glucose disposal would potentially lead to transient hyperglycaemia. By contrast, in lower organisms, one of the consequences of reducing insulin signalling to glucose transport and metabolism is to extend lifespan, suggesting that there may be some potential health benefits of limiting excess glucose transport and metabolism [19, 73, 97, 129, 175, 185]. Some of these beneficial effects are driven by reductions in whole organism calorie intake. Of course, comparisons with simple organism lifespan will over-simplify of the role of glucose transport in human physiology. However, specific targeting of GLUT4 could be explored in animals with a view to it providing better management and rebalancing of glucose homeostasis in conditions where cellular uptake of glucose is excessive.

## Conclusions and perspectives

In recent years, our understanding of GLUT proteins has been greatly enhanced by phylogeny and by the availability of crystal structures of GLUTs in a range of conformations. The crystal structures support the central tenant of the alternating access mode of transport. While this model is consistent with most available data on the kinetics of glucose transport, there remains evidence for a slightly more complex model with the possibility of additional (perhaps rare) states of the protein in which allosteric interactions occur. Recent papers provide a useful discussion of these additional stochastic processes in alternative access modes of membrane transport [85, 99].

The GLUTs1-4, GLUT5 and GLUT13 are well-characterised transporters with often quite clear and separate functions related to their kinetic, specificity and cellular distributions. This group of 6 transporters is already the subject of approaches that aim to generate tools and reagents that can specifically target the individual isoforms, with the goal of generating therapeutics. However, the other 8 GLUTs that make up the family are relatively uncharacterised and importantly, the substrate specificity is in many cases unknown (or in the case of GLUT7 uncertain). Crystal structures of these GLUTs are currently not available but in silico modelling of these GLUT structures based on the GLUT1, 3 and 5 structures will now be possible and may lead to some inferences regarding their likely substrates. Further characterisation of the substrate specificities and clarification of the subcellular distributions of these 8 isoforms will aid in attributing function.

A quantitative approach to defining absolute levels of protein abundance in different tissues is still disappointingly lacking for many GLUTs, and often mRNA levels alone are reported. Using some of the newer protein detection techniques, such as fluorescently tagged protein constructs, it will also be important to establish the extent of regulation of the more recently discovered GLUTs, particularly those of the class 2 and 3. Following methods utilised to study class 1 GLUTs, it would be of interest to define whether there is hormonal and energy-level control of translocation between subcellular compartments. In addition, examination of longer-term changes—including post-translocation palmitoylation, ubiquitinoylation and phosphorylation of the GLUTs and associated proteins, and changes in turnover (transcription and degradation)—will reveal whether these GLUTs are necessary for organism viability and health. In future, with wider human genome sequencing, inherited mutations in these transporters may emerge. Such studies will aid in ascribing function to the class 2 and 3 GLUTs, and thereby increase understanding of any related disease syndromes.

## Data Availability

Not applicable.

## Abbreviations

GGAP2: Golgi associated, gamma adaptin ear containing, ARF-binding protein 2
VAMP: Vesicle-associated membrane protein
IRAP: Insulin-responsive aminopeptidase
UBC9: Polyubiquitin-C precursor 9
USP25: Ubiquitin carboxyl-terminal hydrolase 25
TM: Transmembrane segment
p115: General vesicular transport factor p115
MSNBA: N-[4-(methylsulfonyl)-2-nitrophenyl]-1,3-benzodioxol-5-amine
TBC: Tre-2/Bub2/Cdc16
CHC: Clathrin heavy chain
ERGIC: ER-Golgi intermediate compartment
TGN: Trans-Golgi network
MVB: Multivesicular body
